# Supplementary material for: L1 retrotransposons exploit RNA m6A modification as an evolutionary driving force
Source: Nat Commun. 2021 Feb 9;12:880. doi: 10.1038/s41467-021-21197-1 (PMC7873242; doi:10.1038/s41467-021-21197-1)
Supplement: Supplementary file 1 — Supplementary Information [file 41467_2021_21197_MOESM1_ESM.pdf]

Supplementary Information

L1 retrotransposons exploit RNA m^6A modification as an evolutionary driving force

Hwang *et al.*

Supplementary Methods

Supplementary Figure 1-11

Supplementary Table 1-6

Supplementary Methods

L1-Luciferase retrotransposition assay. Initially, 8×10^4 HeLa cells were plated into 12-well plates. After 18 h, the cells were transfected with 800 ng of L1 plasmid (pYX014) per well using Lipofectamine 3000 (Invitrogen). After 48 h, the medium was exchanged with a medium supplemented with 1 μg/ml of puromycin (Invitrogen). The cells were further selected for 2 days. Luminescence measurement was performed, as described in the section on luciferase assay. In experiments using HeLa cells devoid of eIF3b, cells were not selected with puromycin but were cultivated for 5 days.

Annexin V-APC staining assay. Cells were labeled with 5 μl of APC-conjugated Annexin V (BioLegend, 640920) according to the manufacturer's instructions. After incubation in the dark for 20 min at RT, the cells were analyzed by flow cytometry using Flow-Activated Cell Sorter Canto II (BD Bioscience) and Flowjo software (version 10, Flowjo). The AnnexinV-APC-negative population was regarded as alive, whereas the Annexin V-APC–positive populations were taken as measurements of apoptotic/necrotic cells.

RNA stability assay. HeLa cells were plated at 1.5×10^5 cells per well in a 6-well plate. The following day, the cells were transfected with 1.5 ug of pL1Hs or pL1 m^6A mut using Lipofectamine 3000 (Invitrogen). After 24 h, the cells were re-seeded into 4 wells of a 12-well plate. After 24 h, the cells were treated with 10 μg/ml actinomycin D added at 6, 4, 2, and 0 h before RNA extraction. RNA extraction and RT-qPCR were performed as described in the method section.

Nuclear/Cytosolic fractionation. HeLa cells were plated at 1.5×10^5 cells per well in a 6-well plate. The following day, the cells were transfected with 1 ug of pL1Hs and pL1 m^6A mut using Lipofectamine 3000 (Invitrogen). After 48 h, the cells were fractionated using PARIS kit (Invitrogen) according to the manufacturer's protocol for all steps except the RNA extraction step. RNA was purified using TRIzol reagent and treated with rDNaseI, as described in RNA extraction section.

Quantification of transfected pL1 plasmid Genomic DNA and transfected DNA were purified using the QIAamp DNA Blood Mini Kit (Qiagen) following the manufacturer's instructions. For qPCR analyses of purified DNA, Equivalent amounts of DNA (50 - 100 ng) from each sample were subject to qPCR reaction. Data were normalized to *MDM2*, and L1 plasmid were detected using reporter L1-specific primer. qPCR primers are listed in Supplementary Table 2.

YTHDFs RNA-immunoprecipitation HeLa cells were plated at 1×10^6 cells in 100 mm dishes. The following day, the cells were transfected with 3.5 µg of pL1Hs or m^6A mut and 3.5 µg of HA-YTHDF1 or 2. After 48 h, cells were harvested and resuspended in RIP lysis buffer (150 mM KCl, 10 mM HEPES (pH 7.6), 2 mM EDTA, 0.5% NP40, 1 mM DTT, cOmplete protease inhibitor cocktail (Roche), 400 unit/ ml RNase inhibitor (Enzynomics)) for 10 min at 4°C. Lysates were cleared by centrifugation (4°C, 15,000 × *g* for 15 min) and filtrated by passing through a 0.45-µm membrane syringe filter. Input samples for RNA extraction and immunoblot assay were saved respectively (10% of lysates). For antibody-bead preparation, 4 µl of HA antibody (Cell signaling, 3724) was diluted in 80 µl of NT2 buffer (200 mM NaCl, 50 mM HEPES (pH7.6), 2 mM EDTA, 0.05% NP40) and incubated with Dynabead protein G for 30 min with rotation at room temperature. After incubation, antibody-bead complex were washed and resuspended in a volume of NT2 buffer equivalent to lysate. Lysates were then added into bead-containing tube and incubated overnight on a rotating wheel at 4°C. Afterwards, the beads were subject to wash with 5 × 1 ml portions of ice-cold NT2 buffer. The 20% of beads were resuspended in 40 µl of RIP lysis buffer and analyzed by western blotting with the input samples. The rest 80% of beads as well as saved input RNA samples were mixed with 1 ml of TRIzol (Invitrogen), supplemented with 50 ng of spike-in RNA. RNA was extracted using 1 ml of TRIzol supplemented with 50 ng of spike-in RNA.

Northern blot HeLa cells were seeded in 100 mm dishes at 1×10^6 cells. Next day, 7 µg of reporter-deleted pL1 construct, $pL1Hs^{\Delta BLA}$ or $pL1m^6A\ mut^{\Delta BLA}$ were transfected into cells. At 3 d post transfection, poly (A) + RNA was purified using TRIzol reagent and the Poly (A) purist Mag kit (Invitrogen). The eluted RNA was purified by ethanol precipitation, and dissolved in formaldehyde load dye (Invitrogen). 2 µg of poly (A) + RNA was separated in 1% formaldehyde agarose gels in MOPS gel running buffer (NorthernMax kit, Invitrogen) according to the manufacturer's protocol. After electrophoresis, the lane containing the size markers were cut off and stained separately with EtBr. RNA was then transferred to BrightStar Nylon membranes (Invitrogen) through capillary transfer for 4 h. RNA-bound mebranes were cross-linked using UV light (120 mJ/cm^2) and preincuabated in PerfectHyb hybrydization buffer (NorthernMax kit, Invitrogen) for 30 min at 68°C. Radioisotopes-labeled RNA probes specific to L1 5′ UTR, $Hygromycin^R$, or beta-actin (final concentration $\sim 1 \times 10^6$ cps / ml) were mixed with PerfectHyb hybrydization buffer, and incubated with membrane overnight at

68°C with constant rotation. Membranes were then washed two times with low stringency wash solution (2× SSC, 0.1% SDS) and two times with high stringency wash solution (0.1× SSC, 0.1% SDS). The blots were exposured to Fuji 32P screens, and scanned by Typhoon FLA7000 (version 1.2).

Northern blot probe production RNA probe templates containing T7 promoter sequence were synthesized by PCR. Strand-specific RNA probes were generated and labeled with α-^{32}P UTP using the EZ™ T7 High Yield In Vitro Transcription kit (Enzynomics) according to the manufacturer's instructions. Briefly, 500-800 ng of templates were subject to in vitro transcription reaction (0.5 mM ATP, CTP, GTP, 1 mCi/ml α-^{32}P UTP, 1 mM DTT and 1 unit/μl RNase inhibitor) and incubated for 6 h. After reaction, rDNase I (Takara) was treated to remove template DNA. All probes were purified using NucleoSpin RNA clean-up (MACHEREY-NAGEL). Probe template sequence of L1 5′ UTR, Hygromycin^R, or *beta-actin* are listed in below. T7 promoter sequences are in bold.

L1 5′ UTR (1-232, 232bp)
5′ **TAATACGACTCACTATAGGG**CTCGGAAAGGGAACTCCCTGACCCCTTGCGCTTCCCAGGTG
AGGCAATGCCTCGCCCTGCTTCGGCTCGCGCACGGTGCGCACACACACTGGCCTGCGCCCACTGT
CTGGCACTCCCTAGTGAGATGAACCCGGTACCTCAGATGGAAATGCAGAAATCACCGTCTTCTG
CGTCGCTCACGCAGGGAGCTGTAGACCGGAGCTGTTCCTATTCGGCCATCTTGGCTCCTCCC 3′

Hygromycin^R (181-326, 146bp)
5′ **TAATACGACTCACTATAGGG**CGTTATGTTTATCGGCACTTTGCATCGGCCGCGCTCCCGAT
TCCGGAAGTGCTTGACATTGGGGAATTCAGCGAGAGCCTGACCTATTGCATCTCCCGCCGTGCAC
AGGGTGTCACGTTGCAAGACCTGCCTGAAACCGAACTGCC 3′

beta-actin (1464-1588, 125bp)
5′ **TAATACGACTCACTATAGGG**CCAAATATGAGATGCGTTGTTACAGGAAGTCCCTTGCCATC
CTAAAAGCCACCCCACTTCTCTCTAAGGAGAATGGCCCAGTCCTCTCCCAAGTCCACACAGGGG
AGGTGATAGCATTGCTTTCG 3′

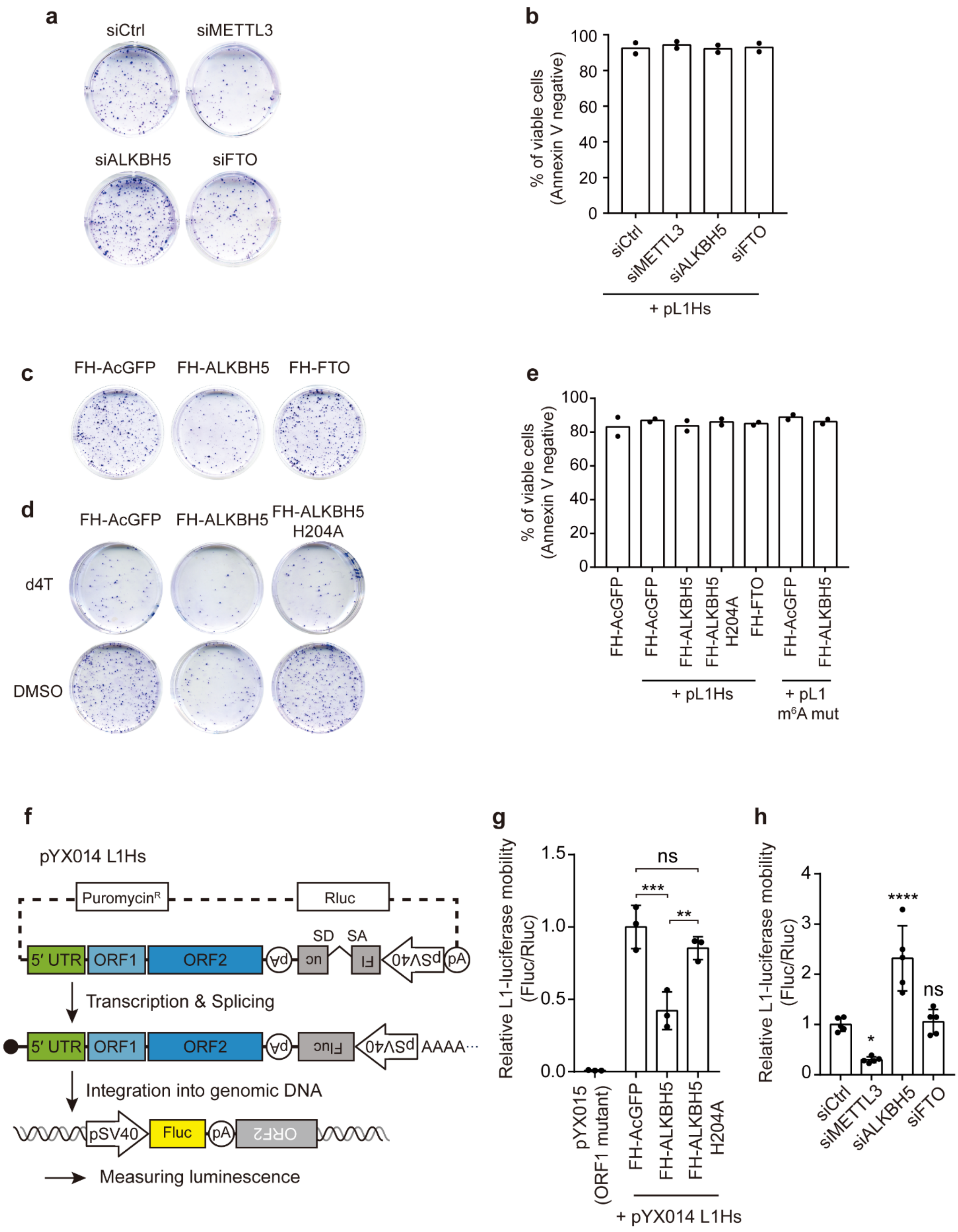
a
siCtrl
siMETTL3
siALKBH5
siFTO
b
% of viable cells (Annexin V negative)
siCtrl
siMETTL3
siALKBH5
siFTO
+ pL1Hs
c
FH-AcGFP
FH-ALKBH5
FH-FTO
d
FH-AcGFP
FH-ALKBH5
FH-ALKBH5 H204A
d4T
DMSO
e
% of viable cells (Annexin V negative)
FH-AcGFP
FH-AcGFP
FH-ALKBH5
FH-ALKBH5 H204A
FH-FTO
FH-AcGFP
FH-ALKBH5
+ pL1Hs
+ pL1 m6A mut
f
pYX014 L1Hs
PuromycinR
Rluc
5′ UTR
ORF1
ORF2
pA
SD
SA
pSV40
Transcription & Splicing
Fluc
AAAA···
Integration into genomic DNA
Measuring luminescence
g
Relative L1-luciferase mobility (Fluc/Rluc)
ns
***
**
pYX015 (ORF1 mutant)
FH-AcGFP
FH-ALKBH5
FH-ALKBH5 H204A
+ pYX014 L1Hs
h
Relative L1-luciferase mobility (Fluc/Rluc)
****
*
ns
siCtrl
siMETTL3
siALKBH5
siFTO

Supplementary Figure 1. RNA methylation machinery controls L1 retrotransposition.
(a) Representative images of blasticidin S-resistant HeLa colonies from the L1 retrotransposition assay (related to Figure 1b). **(b)** Annexin V assay of pL1Hs-expressing HeLa cells treated with siRNA that targets METTL3, ALKBH5, or FTO. (related to Figure 1b, n = 2 independent samples, mean of two replicates) **(c and d)** Representative pictures of L1 blasticidin S retrotransposition assay. (related to Figure 1c and d, n = 2 independent samples, mean of two replicates) **(e)** Annexin V assay of pL1-expressing HeLa cells which are co-transfected with plasmids-encoding indicated genes. (related to Figure 1c, d, and 3f, n = 2 independent samples, mean of two replicates) **(f)** A schematic of the L1-luciferase reporter construct (pYX014) used in this study. This construct drives L1-luciferase expression using its promoter in the 5′ UTR. Firefly luciferase acts as the L1 retrotransposition reporter, while *Renilla* luciferase is used for the normalization of transfection efficiency. **(g)** L1-luciferase retrotransposition assays performed in ALKBH5 or ALKBH5 ^{H204A} –overexpressing HeLa cells. In the dual-luciferase assay, the levels of firefly luciferase were measured using the luminescence of *Renilla* luciferase. The pYX015 L1 construct carries retrotransposition-defective ORF1 mutations. The ratio of the luminescence of firefly and *Renilla* luciferase (Fluc/Rluc) was normalized to that in AcGFP-overexpressing cells, which served as a control. **(h)** L1-luciferase retrotransposition assay using m^6A enzyme-depleted HeLa cells. L1 mobility assessment was performed as in (g). (three (g) or five (h) independent samples, mean ± s.d., one-way ANOVA with Tukey's (g) or Dunnett's multiple comparisons test (h); ****p < 0.0001, ***p < 0.001, **p < 0.01 and *p < 0.05, in comparison to control, ns = not significant). Source data are provided as a Source data file.

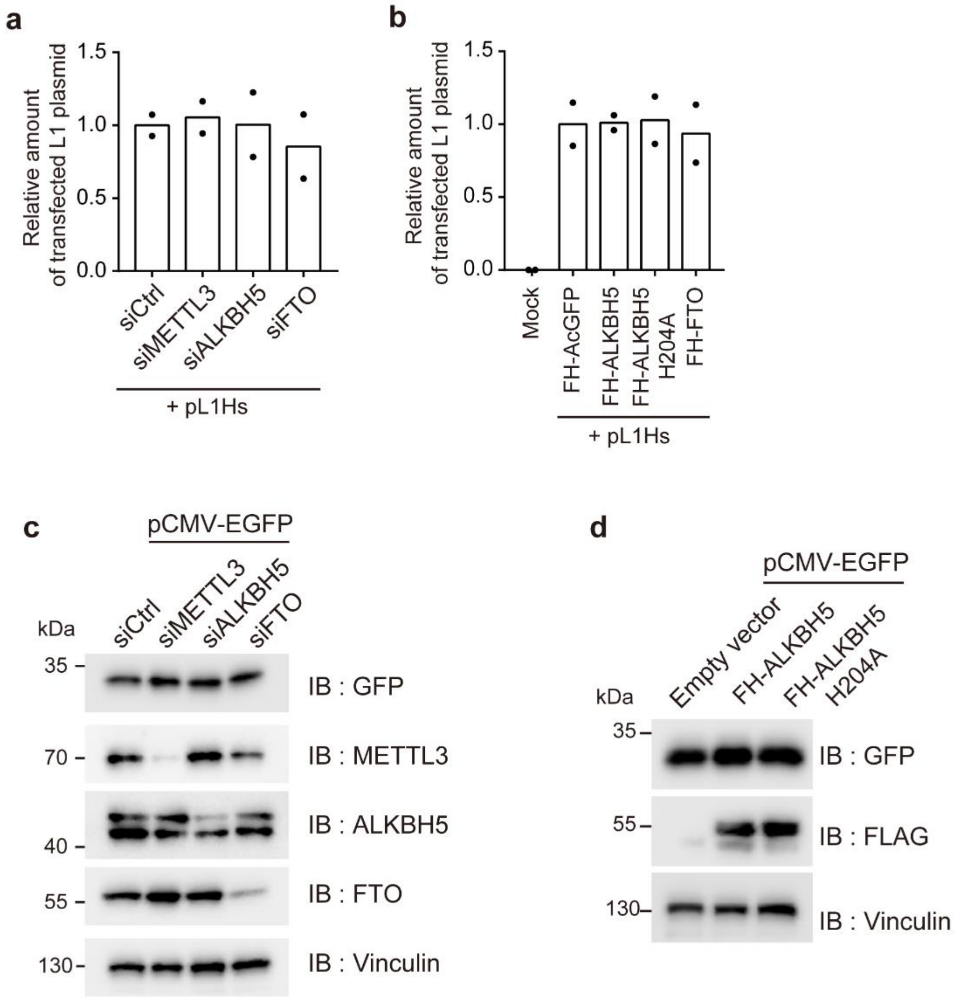

Supplementary Figure 2. Modulation of RNA m6A enzymes expression did not affect transfection efficiency. **(a and b)** Quantification of transfected pL1 amount in HeLa cells transfected with indicated siRNAs that target m^{6}A enzymes (a) or with plasmids-encoding indicated RNA demethylases (b). Extracted gDNA and L1 plasmids were analyzed by qPCR. The enrichment of transfected pL1 was normalized to the levels of *MDM2*. (related to Figure 1e, f and g, n = 2 independent samples, mean of two replicates) **(c and d)** EGFP expression test after indicated siRNA treatment (c) or co-transfection with indicated plasmids (d). EGFP expression was measured for determining the transfection efficiency of pCMV-EGFP and the translation of exogenous gene. The protein ladder is marked with the corresponding molecular weight. Vinculin served as a loading control. The immunoblot images (c and d) are representative of two independent experiments. Source data are provided as a Source data file.

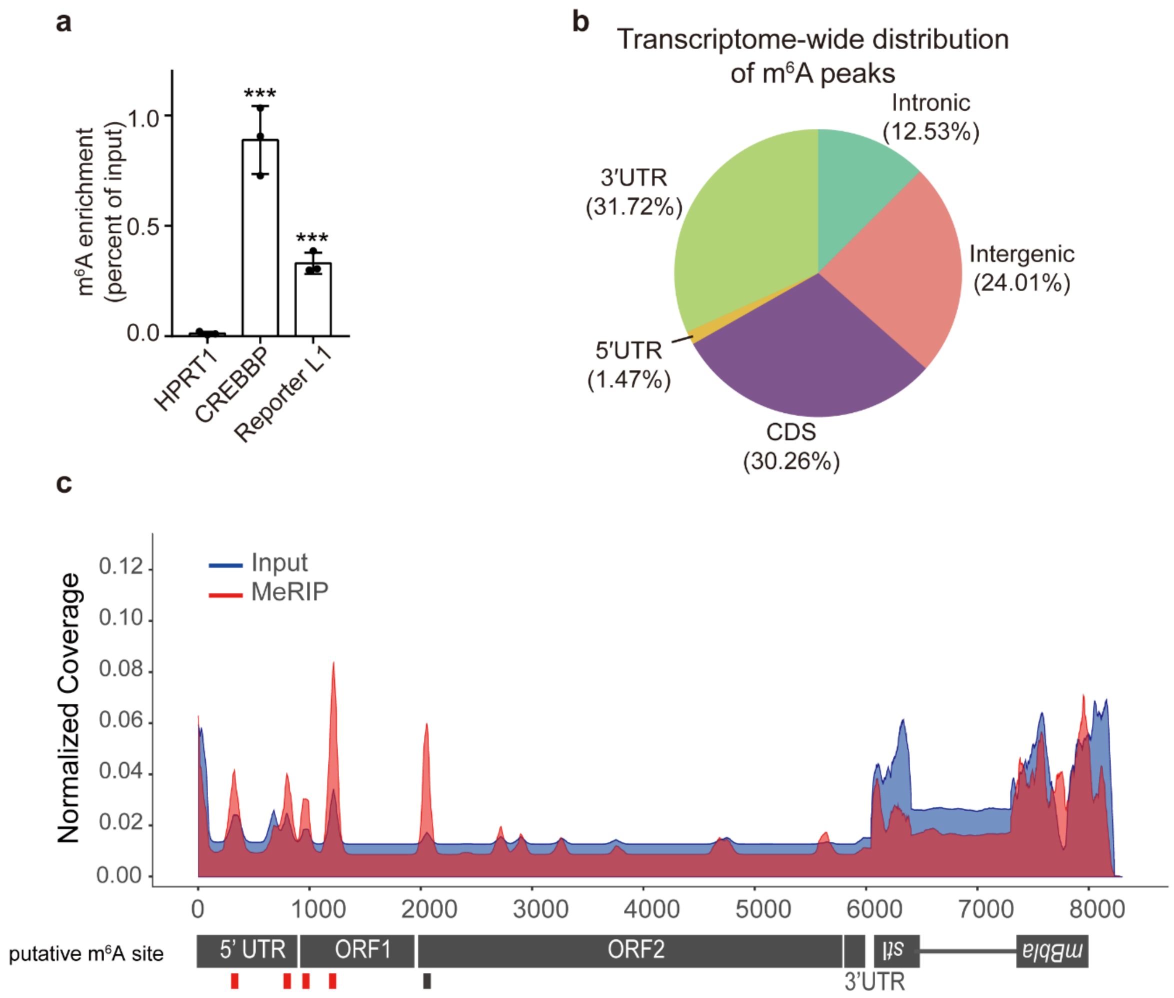

Supplementary Figure 3. L1 RNA is modified by m^{6}A. **(a)** MeRIP-qPCR assay of pL1Hs-expressing HeLa cells. The enrichment of the m^{6}A antibody-bound RNA was calculated as a percentage of the input. (n = 3 independent samples, mean ± s.d., unpaired two-tailed t test; ***p < 0.001) **(b)** Transcriptome-wide distribution of m^{6}A peaks from MeRIP-seq data of pL1Hs-expressing HeLa cells. Pie charts indicate the percentage of m^{6}A peaks in the marked region. m^{6}A is highly enriched in 3′ UTR and CDS compared to the distribution of reads in the input samples. **(c)** Methylation peaks in the full-length pL1Hs containing a retrotransposition reporter. The pL1Hs construct is presented below. *mblast*I, the reporter, is antisense and the gamma-globin intron is inserted in it, as described in Fig. 1A. Fragmented poly(A) RNA from pL1Hs-expressing HeLa cells was subject to RNA-seq and MeRIP-seq, and analyzed as described previously[1]. The plot in blue corresponds to the mapping distribution of the input RNA-seq data, while the plot in red corresponds to the MeRIP-seq data. Source data are provided as a Source data file.

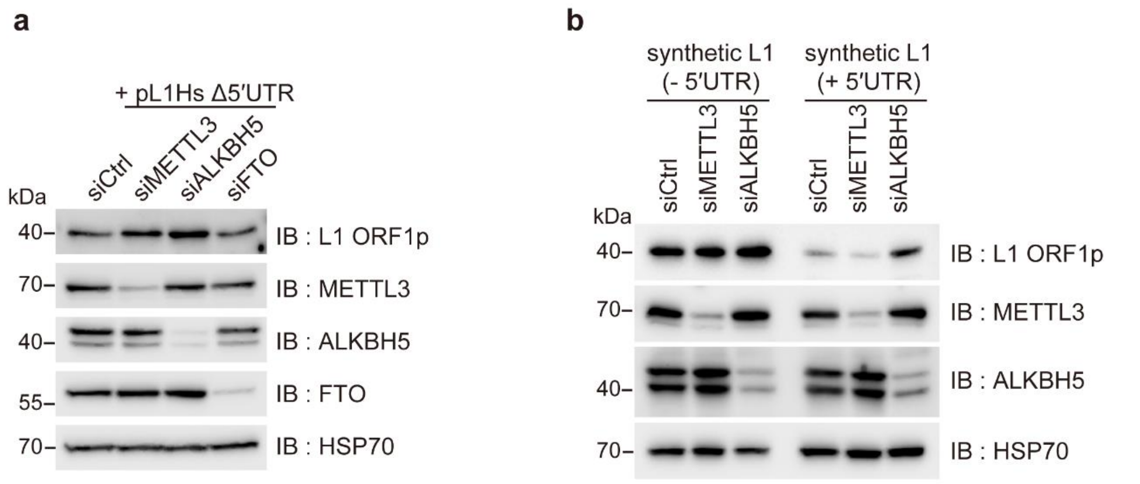

Supplementary Figure 4. L1 5′ UTR m^{6}A cluster promotes L1 activity. **(a)** Immunoblot assay of 5′ UTR deletion L1 construct (pL1Hs Δ5′ UTR) in HeLa cells treated with indicated siRNAs. **(b)** Immunoblot assay depicting the effect of 5′ UTR using synthetic L1 constructs (L1-neo-TET). siRNA and plasmids transfection were performed as in (a). HSP70 served as a loading control. The immunoblot images (a and b) are representative of two independent experiments. Source data are provided as a Source data file.

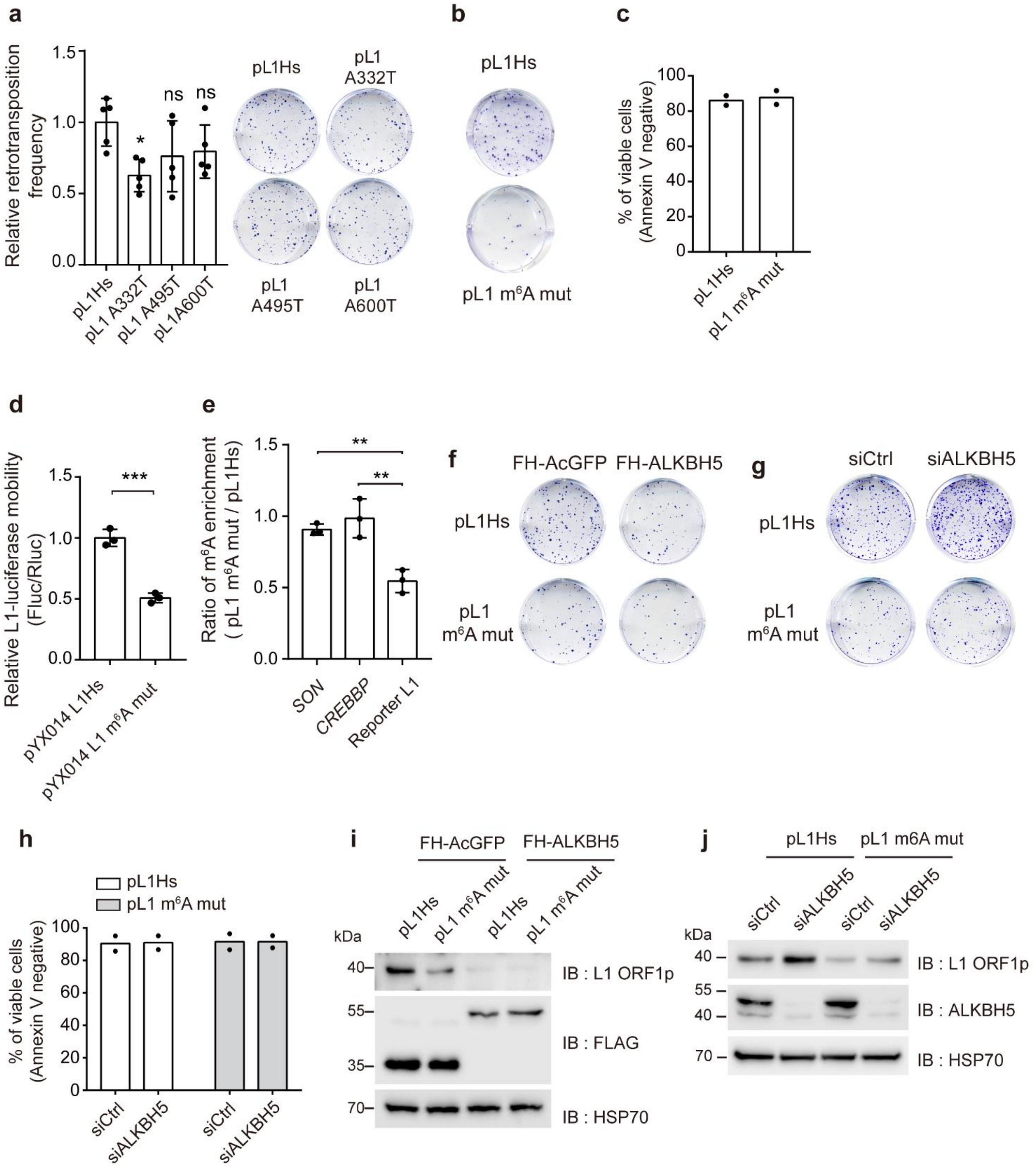
a
Relative retrotransposition frequency
pL1Hs
pL1 A332T
pL1 A495T
pL1A600T
ns
ns
*
pL1 A332T
pL1 A495T
pL1 A600T
b
pL1Hs
pL1 m6A mut
c
% of viable cells (Annexin V negative)
pL1Hs
pL1 m6A mut
d
Relative L1-luciferase mobility (Fluc/Rluc)
***
pYX014 L1Hs
pYX014 L1 m6A mut
e
Ratio of m6A enrichment (pL1 m6A mut / pL1Hs)
**
**
SON
CREBBP
Reporter L1
f
FH-AcGFP
FH-ALKBH5
pL1Hs
pL1 m6A mut
g
siCtrl
siALKBH5
pL1Hs
pL1 m6A mut
h
pL1Hs
pL1 m6A mut
% of viable cells (Annexin V negative)
siCtrl
siALKBH5
siCtrl
siALKBH5
i
FH-AcGFP
FH-ALKBH5
pL1Hs
pL1 m6A mut
pL1Hs
pL1 m6A mut
kDa
IB : L1 ORF1p
IB : FLAG
IB : HSP70
j
pL1Hs
pL1 m6A mut
siCtrl
siALKBH5
siCtrl
siALKBH5
kDa
IB : L1 ORF1p
IB : ALKBH5
IB : HSP70

Supplementary Figure 5. (a) Retrotransposition assay with a pL1Hs construct with a single m^{6}A mutation. The retrotransposition frequency was normalized to that of pL1Hs. Representative images of retrotransposition-positive HeLa foci are shown to the right of the bar graph (n = 5 independent samples, mean ± s.d., one-way ANOVA and Dunnett's multiple comparisons test; *p < 0.05, in comparison to control, ns = not significant). **(b)** Representative pictures of L1 blasticidin S retrotransposition assay (related to Figure 3d). **(c)** Viability test of pL1Hs- or pL1 m^{6}A mut-expressing HeLa cells through Annexin V assay (related to Figure 3d, n = 2 independent samples, mean of two replicates). **(d)** L1-luciferase retrotransposition assay using pYX014 L1Hs- or its m^{6}A mut. Ratios of luminescence (Fluc/Rluc) were normalized to those of pYX014 L1Hs. (n = 3 independent samples, mean ± s.d., unpaired two-tailed t test; ***p < 0.001) **(e)** The ratio of m^{6}A enrichment was calculated using MeRIP-qPCR with pL1Hs- or pL1m^{6}A mut-expressing HeLa cells. *SON* and *CREBBP* served as a transfection-independent control. (n = 3 independent samples, mean ± s.d., one-way ANOVA and Tukey's multiple comparisons test; **p < 0.01, in comparison to reporter L1) **(f and g)** Representative pictures of L1 retrotransposition assay (related to Figure 3f and g). **(h)** Annexin V viability test of pL1Hs- or pL1 m^{6}A mut-expressing HeLa cells which is treated with indicated siRNAs. (related to Figure 3g, n = 2 independent samples, mean of two replicates) **(i)** Immunoblot assay for ORF1p quantification using HeLa cells co-transfected with pL1 construct and AcGFP- or ALKBH5-encoding plasmids. (The predicted molecular weight; 34 kDa for FH-AcGFP and 51 kDa for FH-ALKBH5) **(j)** Immunoblot assay depicting L1 ORF1p expression of indicated pL1-expressing HeLa cells with knockdown of ALKBH5. The non-targeting siRNA siCtrl served as a control. HSP70 served as a loading control. The immunoblot images (i and j) are representative of three independent experiments. Source data are provided as a Source data file.

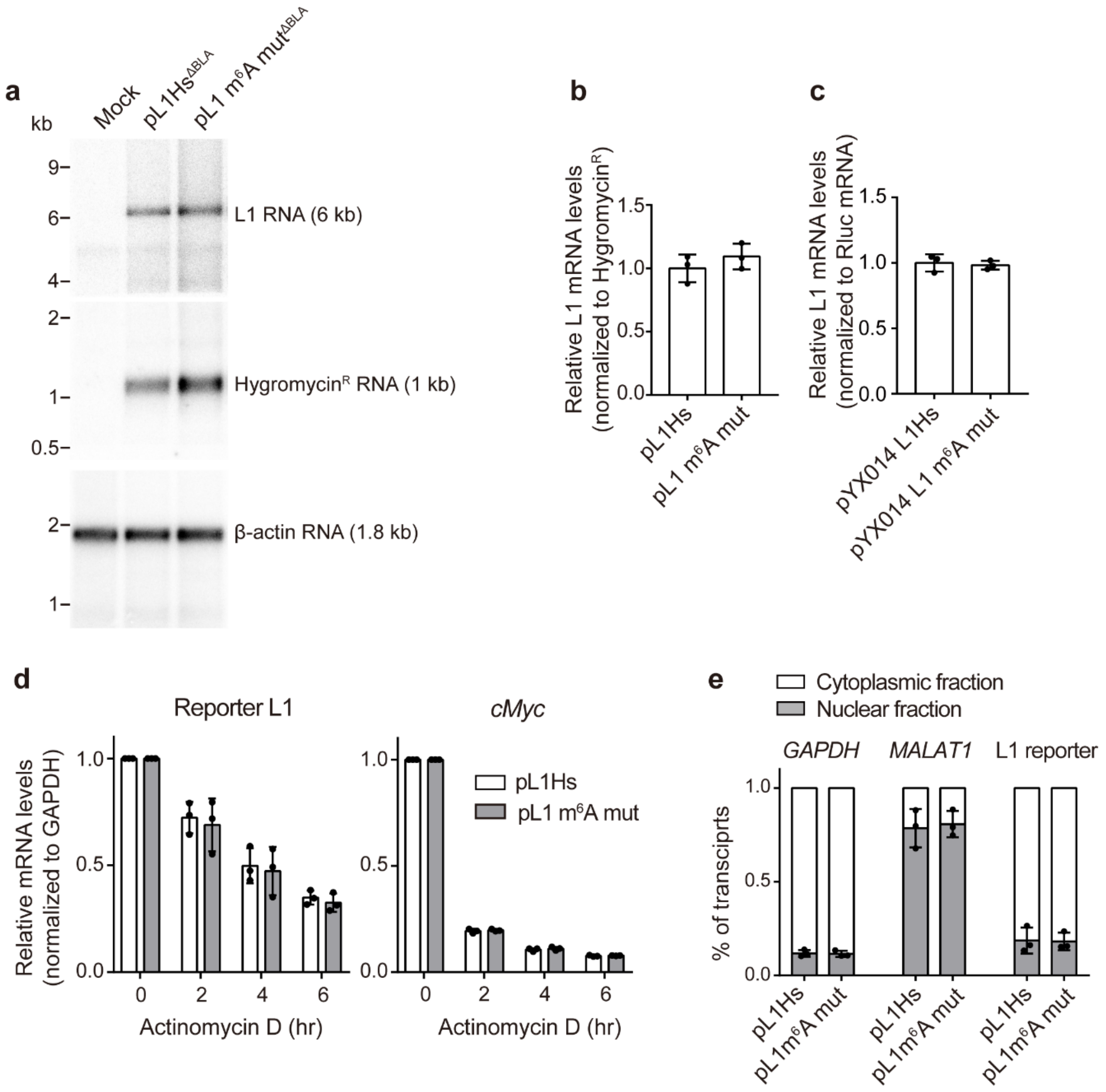

Supplementary Figure 6. L1 5′ UTR m^{6}A cluster does not affect RNA expression, stability, and cellular localization. **(a)** Northern blot of full-length L1 mRNAs expressed from a plasmid encoding a full-length L1Hs or L1 m^{6}A mut lacking a reporter cassette (pL1Hs$^{\Delta BLA}$ or pL1m^{6}A mut$^{\Delta BLA}$). Hygromycin^R and β-actin served as loading controls. Marks on the left indicate positions of the RNA reference. Northern blot images are representative of two independent experiments. **(b and c)** The levels of RNA expression of reporter L1. HeLa cells were transfected with pL1Hs and its m^{6}A mutant construct (b), or pYX014 L1Hs and its mutant (c). The relative levels of reporter L1 transcripts are normalized to those of pL1Hs-encoded hygromycin-resistant gene (b) or pYX014-encoded *Renilla* luciferase mRNA (c). (n = 3 independent samples, mean ± s.d., unpaired two-tailed t test; p = 0.3403 for (b) and p = 0.7003 for (c)) **(d)** L1 RNA decay assay using pL1Hs-or pL1 m^{6}A mut-expressing HeLa cells. The cells were harvested at 0, 2, 4, and 6 h after actinomycin D treatment. mRNA levels were normalized to those of *GAPDH*. *cMyc* served as a positive control for this assay. (n = 3 independent samples, mean ± s.d., two-way ANOVA; p = 0.5003 for reporter L1 and p = 0.2826 for *cMyc*) **(e)** Distribution of reporter L1 RNA in the cytoplasmic and nuclear fraction of pL1-expressing HeLa cells. The percentage of transcripts was estimated assuming that the sum of the percentages of cytoplasmic and nuclear transcripts is 100%. (n = 3 independent samples, mean ± s.d., unpaired two-tailed t test; p = 0.8794 for *GAPDH*, p = 0.7721 for *MALAT1*, p = 0.9235 for reporter L1). Source data are provided as a Source data file.

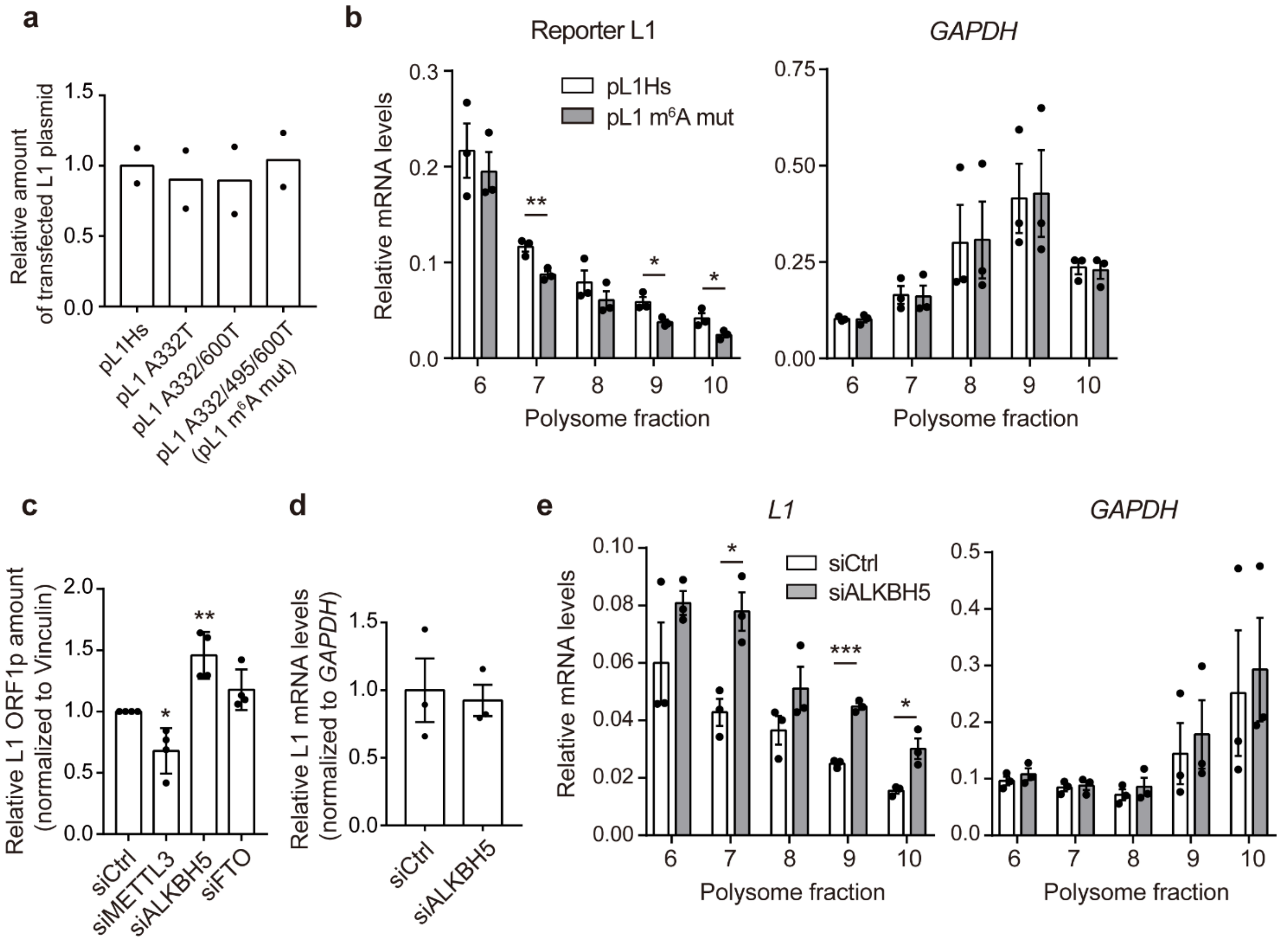

Supplementary Figure 7. m^{6}A modification at L1 5′ UTR cluster enhances the translational efficiency of L1 RNA. **(a)** Quantification of transfected pL1 amount in HeLa cell expressing indicated pL1 constructs. (related to Figure 4a, n = 2 independent samples, mean of two replicates) **(b)** Relative mRNA levels of polysome-bound reporter L1 and *GAPDH*. Polysome profiling of pL1-expressing HeLa cells was performed by sucrose gradient sedimentation. Specific mRNA levels were measured using RT-qPCR. The values are normalized to those of spike-in RNA and then to those of input RNA. (n = 3 independent samples, mean ± s.e.m., unpaired two-tailed t test; **p < 0.01, *p < 0.05) **(c)** Quantification of L1 ORF1p levels of Figure 4c. L1 ORF1p amounts are normalized to those of Vinculin. (n = 4 independent samples, mean ± s.d., Dunnett's multiple comparisons test; **p < 0.01, *p < 0.05) **(d)** Endogenous L1 mRNA expression levels in ALKBH5-depleted PA-1 cells. The RNA levels were estimated using RT-qPCR with the specific primer for *L1* 5′ UTR and *GAPDH*. (n = 3 independent samples, mean ± s.e.m., unpaired two-tailed t test; p = 0.7852) **(e)** Relative mRNA levels of polysome-bound endogenous L1 RNA from ALKBH5-depleted PA-1 cells. The polysome-bound RNA was quantified as in (b). L1 5′ UTR-specific primer was used to detect the endogenous L1 RNA. RNA expression levels were first normalized to those of spike-in RNA and then to those of input RNA (n = 3 independent samples, mean ± s.e.m., unpaired two-tailed t test; ***p < 0.001, *p < 0.05). Source data are provided as a Source data file.

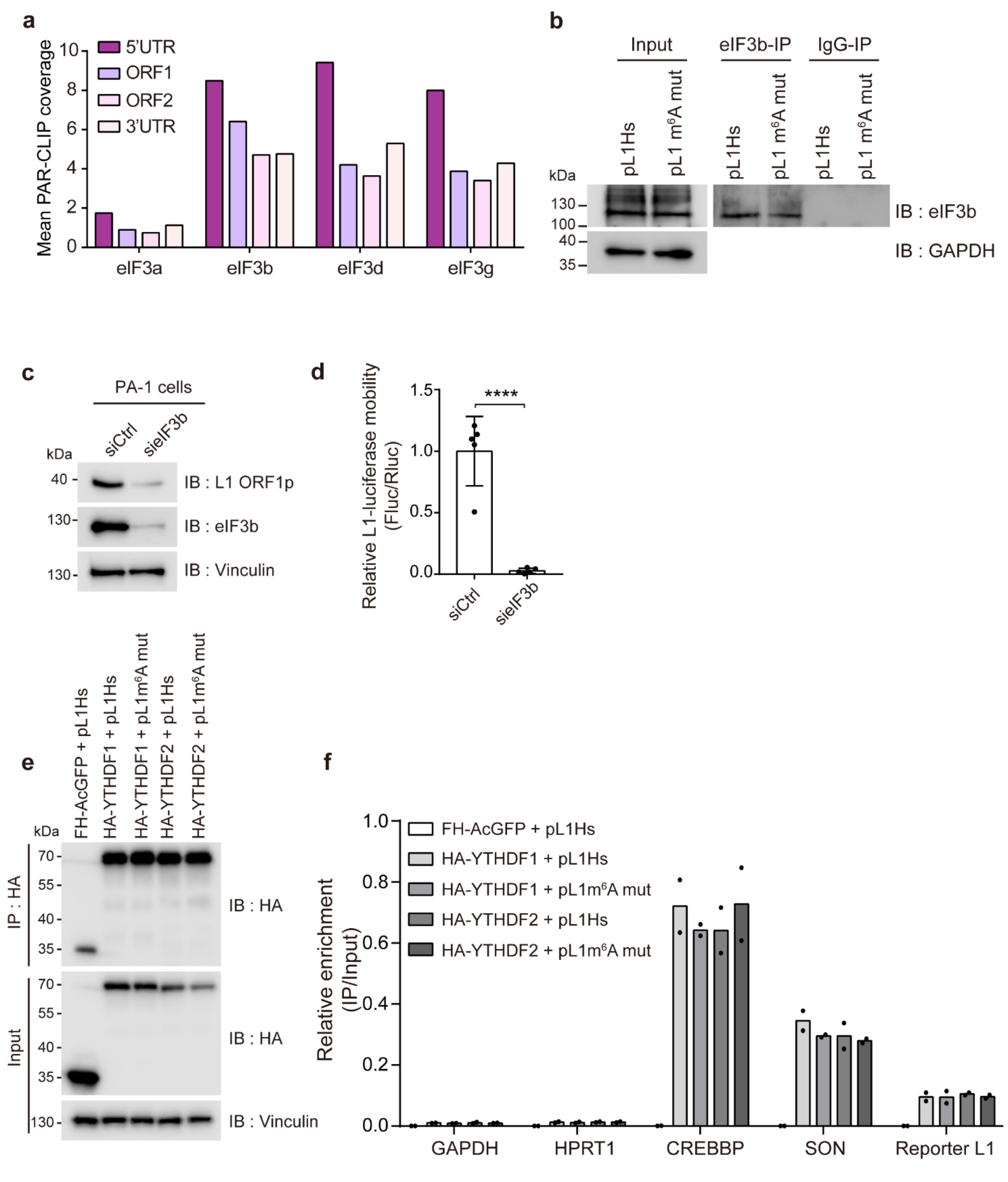
a
Mean PAR-CLIP coverage
5'UTR
ORF1
ORF2
3'UTR
eIF3a
eIF3b
eIF3d
eIF3g
b
Input
eIF3b-IP
IgG-IP
pL1Hs
pL1 m6A mut
kDa
IB : eIF3b
IB : GAPDH
c
PA-1 cells
siCtrl
sieIF3b
IB : L1 ORF1p
IB : eIF3b
IB : Vinculin
d
Relative L1-luciferase mobility (Fluc/Rluc)
****
e
FH-AcGFP + pL1Hs
HA-YTHDF1 + pL1Hs
HA-YTHDF1 + pL1m6A mut
HA-YTHDF2 + pL1Hs
HA-YTHDF2 + pL1m6A mut
IP : HA
Input
IB : HA
IB : Vinculin
f
Relative enrichment (IP/Input)
GAPDH
HPRT1
CREBBP
SON
Reporter L1

Supplementary Figure 8. The m[6]A cluster at L1 5′ UTR acts as a docking site for eIF3. **(a)** Distribution of eIF3 subunit crosslinking sites along the L1Hs consensus sequence. Previously reported data (GSE65004) was used for the analysis. **(b)** Immunoblot assay of eIF3b-immunoprecipitation samples. HeLa cells were transfected with pL1 constructs. Two days after transfection, the cells were subject to UV crosslinking and eIF3b immunoprecipitation. The samples were boiled after adding 10 nM $MgCl_2$ to fragment the crosslinked RNA. GAPDH served as the loading control for the input lysates. The immunoblot images are representative of three independent experiments. **(c)** Immunoblot assay of PA-1 cells devoid of eIF3b by using siRNA. Vinculin served as a loading control. The immunoblot images are representative of two independent experiments. **(d)** L1-luciferase retrotransposition assay using pYX014 L1Hs constructs. pYX014 L1Hs was transfected into sieIF3b-treated HeLa cells. After 5 d from transfection, luminescence of firefly luciferase (Fluc) and of renilla luciferase (Rluc) were measured. Ratios of luminescence (Fluc/Rluc) were normalized to those of siCtrl ($n = 5$ independent samples, mean ± s.d., unpaired two-tailed t test; ****$p < 0.0001$). **(e and f)** RNA-immunoprecipitation assay of HA-YTHDF and pL1 co-transfected HeLa cells. Immunoblot assay of HA-immunoprecipitation samples (e). RNA-IP qPCR of HA-immunoprecipitation eluates (f). Immunoprecipitation using FH-AcGFP served as negative control. The levels of RNA were normalized to the spike-in control and to the levels of input RNA ($n = 2$ independent samples, mean of two replicates). The immunoblot images of (e) are representative of two independent experiments. Source data are provided as a Source data file.

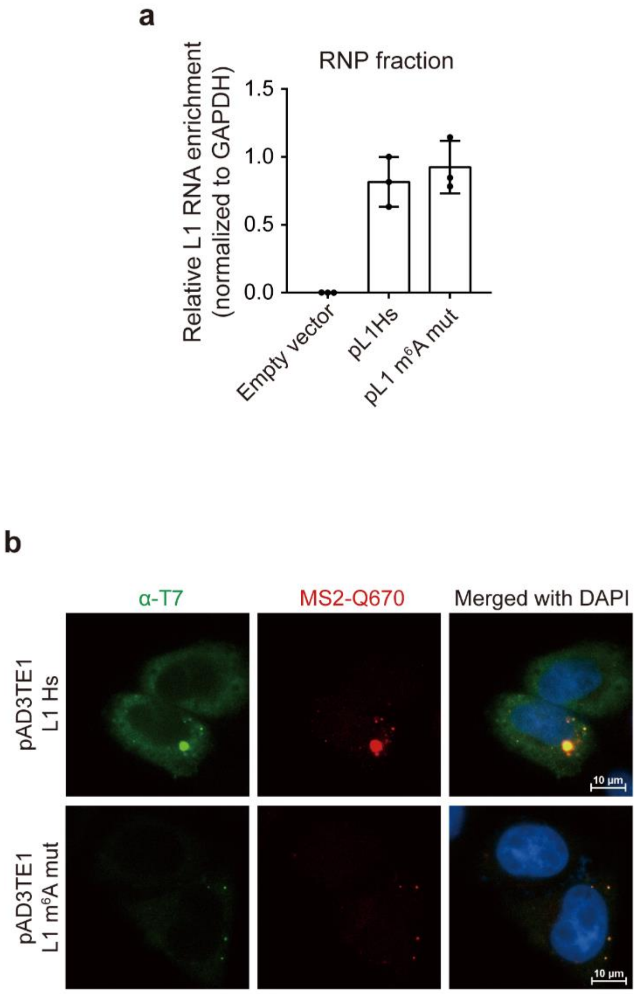

Supplementary Figure 9. m^{6}A modification is crucial for generating retrotransposition-competent L1 RNPs. **(a)** RT-qPCR analysis using the purified RNP fraction of pL1-expressing HeLa cells. The levels of L1 RNA were normalized to the spike-in control and *GAPDH* mRNA (n = 3 independent experiments, mean ± s.d.). **(b)** Nonsaturated version of Figure 5f. Images for T7-tagged ORF1p (green), L1-MS2 RNA (red), and the merged images with DAPI (blue) are indicated. The images are representative of two independent experiments. Source data are provided as a Source data file.

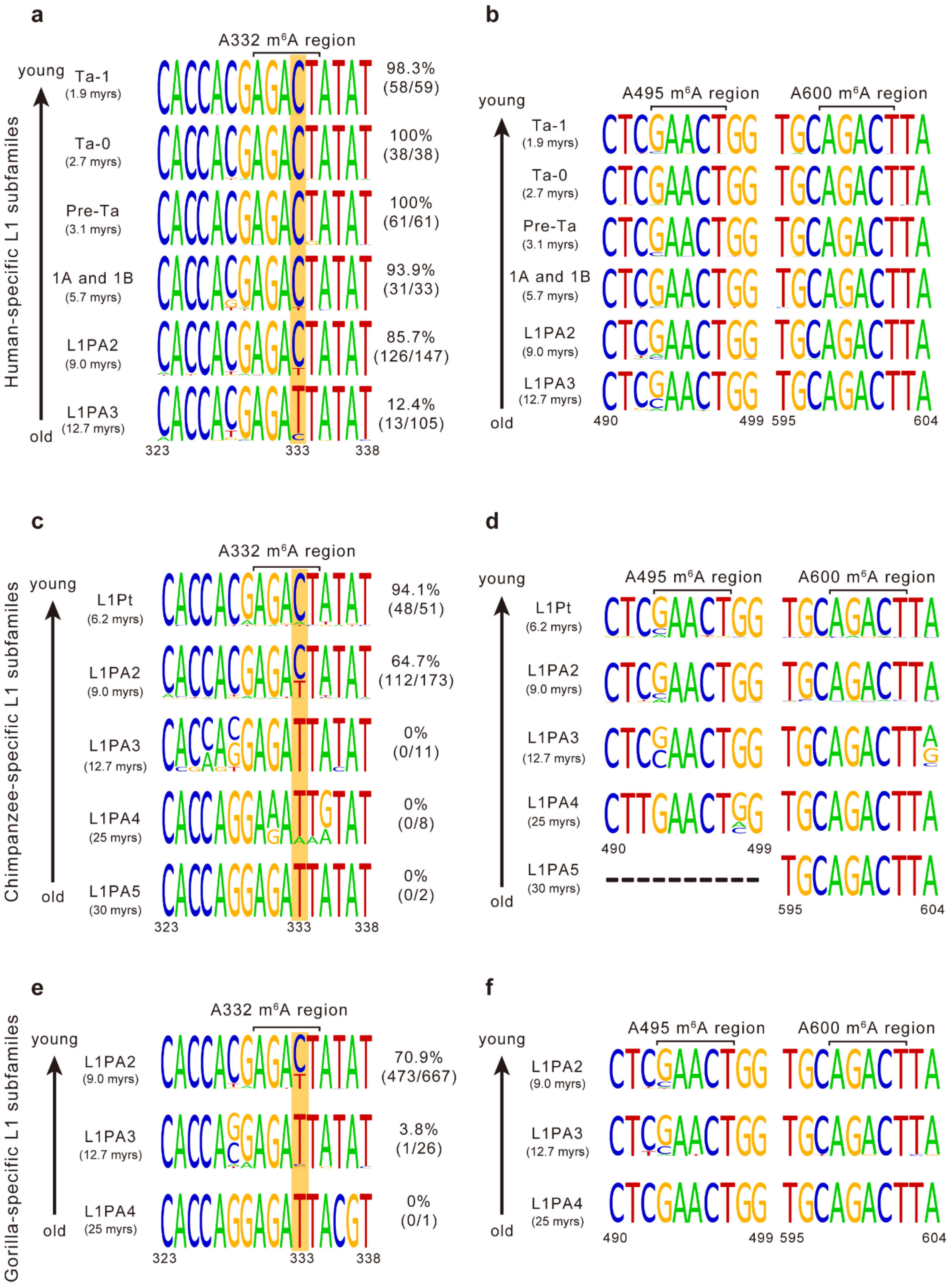
a
A332 m6A region
young
old
Human-specific L1 subfamiles
Ta-1 (1.9 myrs) 98.3% (58/59)
Ta-0 (2.7 myrs) 100% (38/38)
Pre-Ta (3.1 myrs) 100% (61/61)
1A and 1B (5.7 myrs) 93.9% (31/33)
L1PA2 (9.0 myrs) 85.7% (126/147)
L1PA3 (12.7 myrs) 12.4% (13/105)
323
333
338
b
A495 m6A region
A600 m6A region
young
old
Ta-1 (1.9 myrs)
Ta-0 (2.7 myrs)
Pre-Ta (3.1 myrs)
1A and 1B (5.7 myrs)
L1PA2 (9.0 myrs)
L1PA3 (12.7 myrs)
490
499
595
604
c
A332 m6A region
young
old
Chimpanzee-specific L1 subfamiles
L1Pt (6.2 myrs) 94.1% (48/51)
L1PA2 (9.0 myrs) 64.7% (112/173)
L1PA3 (12.7 myrs) 0% (0/11)
L1PA4 (25 myrs) 0% (0/8)
L1PA5 (30 myrs) 0% (0/2)
323
333
338
d
A495 m6A region
A600 m6A region
young
old
L1Pt (6.2 myrs)
L1PA2 (9.0 myrs)
L1PA3 (12.7 myrs)
L1PA4 (25 myrs)
L1PA5 (30 myrs)
490
499
595
604
e
A332 m6A region
young
old
Gorilla-specific L1 subfamiles
L1PA2 (9.0 myrs) 70.9% (473/667)
L1PA3 (12.7 myrs) 3.8% (1/26)
L1PA4 (25 myrs) 0% (0/1)
323
333
338
f
A495 m6A region
A600 m6A region
young
old
L1PA2 (9.0 myrs)
L1PA3 (12.7 myrs)
L1PA4 (25 myrs)
490
499
595
604

Supplementary Figure 10. Comparative analysis of the m^6A cluster in speciec-specific full-length L1 subfamilies of three different primates. **(a)** Comparative analysis of the A332 m^6A region (A323-A338) in human-specific full-length L1 subfamilies. **(b)** Comparative analysis of other m^6A regions at the A495 and the A600 residues in human-specific full-length L1 subfamilies (left: A495, right: A600). Differences in the substitution patterns in different subfamilies were not observable at the two other m^6A sites. **(c)** Comparative analysis of the A332 m^6A region (A323-A338) in chimpanzee-specific full-length L1 subfamilies. **(d)** Comparative analysis of other m^6A regions at the A495 and the A600 residues in chimpanzee-specific full-length L1 subfamilies (left: A495, right: A600). The sequence reads of the A495 region were undefined in L1PA5 subfamilies. Differences in the substitution patterns in different subfamilies were not observable at the two other m^6A sites. **(e)** Comparative analysis of the A332 m^6A region (A323-A338) in gorilla-specific full-length L1 subfamilies. **(f)** Comparative analysis of other m^6A regions at the A495 and the A600 residues in gorilla-specific full-length L1 subfamilies (left: A495, right: A600). Differences in the substitution patterns in different subfamilies were not observable at the two other m^6A sites. In (a), (c), and (e), the yellow box indicates the T to C substitution site at the 333rd residue. The percentage indicates the proportion of m^6A positive L1s to total L1s. The age of the L1 lineage is specified in parentheses. The height of the nucleotide indicates the frequency of the corresponding nucleotide.

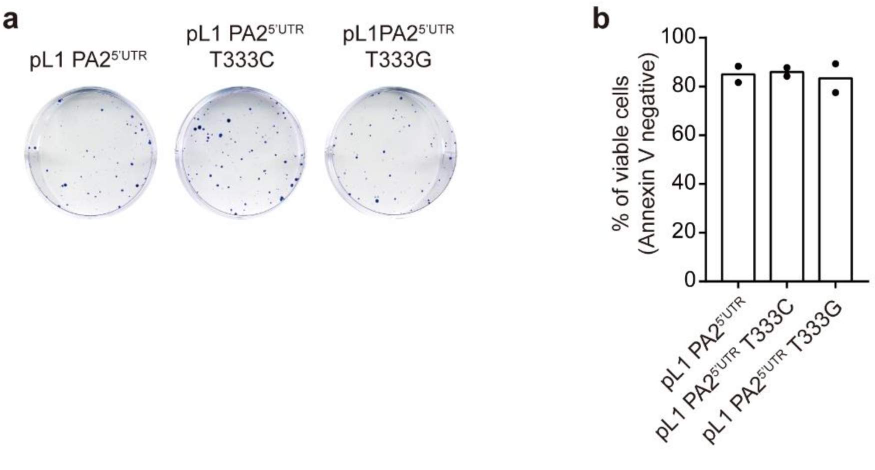

Supplementary Figure 11. L1 retrotransposition assay using L1PA2 and its mutant constructs, T333C and T333G. **(a)** Representative images of blasticidin S-resistant HeLa colonies from the L1 retrotransposition assay (related to Figure 6c). **(b)** Viability test of pL1 PA2$^{5'UTR}$- or its mutant constructs-expressing HeLa cells through Annexin V assay (related to Figure 6d, n = 2 independent samples, mean of two replicates). Source data are provided as a Source data file.

a

hESC MeRIP-seq

#	m^6A peak location	Feature	SRAMP prediction score: high score position	SRAMP prediction score: moderate score position
1	171-241	5'UTR	none	none
2	266-336	5'UTR	332	none
3	379-449	5'UTR	none	410
4	459-529	5'UTR	495	none
5	532-602	5'UTR	569	581
6	570-640	5'UTR	600	none
7	631-701	5'UTR	679	703
8	713-783	5'UTR	none	758
9	806-876	5'UTR	839	none
10	974-1044	ORF1	none	none
11	2228-2298	ORF2	none	2234
12	3972-4042	ORF2	none	none
13	4954-5024	ORF2	none	none
14	5225-5295	ORF2	none	none
15	5437-5507	ORF2	none	none
16	5601-5671	ORF2	none	none
17	5829-5899	3'UTR	none	none
18	5983-6018	3'UTR	none	none

b

pL1Hs-transfected HeLa MeRIP-seq

#	m^6A peak location	Feature	SRAMP prediction score: high score position	SRAMP prediction score: moderate score position
1	293-363	5'UTR	332	none
2	800-870	5'UTR	839	758
3	899-969	ORF1	931	none
4	1206-1276	ORF1	1203	none
5	1998-2068	ORF2	2037, 2064	none

Supplementary Table 1. Identification of putative m^6A peaks through MeRIP-seq. **(a)** Identification of endogenous L1 m^6A peaks by hESC MeRIP-seq. The peaks were identified by manual inspection. The ratio of read coverage (MeRIP/input) from two replicates was measured. The sections with average ratio > 1.3 were selected, and the ± 35 nt regions from the maximum value were assumed to represent m^6A peaks. The 18 identified peaks and their respective features are indicated. The SRAMP m^6A tool was used to detect putative m^6A sites in the peaks. Both high and moderate score positions in the corresponding peaks were noted. Peaks with high score prediction sites were marked in apricot. **(b)** Identification of m^6A peaks in reporter L1 through MeRIP-seq of pL1Hs-expressing HeLa. Peaks were identified as in (a), although the cut-off ratio was > 1.4. SRAMP was also conducted to determine the putative m^6A regions, as in (a). Peaks in apricot indicate high score prediction.

qPCR and RT-PCR primer

HPRT1	
F	TGA CAC TGG CAA AAC AAT GCA
R	GGT CCT TTT CAC CAG CAA GCT

SON	
F	TGA CAG ATT TGG ATA AGG CTC A
R	GCT CCT CCT GAC TTT TTA GCA A

CREBBP	
F	CAT GGC CAA GAT GGG AAT AA
R	TGC ATC TGA GAC ATA TTT GGC

L1 5'UTR	
F	AGC CTA ACT GGG AGG CA
R	TTC CCC ATC TTT GTG GT

L1 ORF1	
F	GCA AGG CAG GCC AAC GTT CA
R	CCT TTC TCT CTG GCT GCC CT

L1 ORF2	
F	CAG GGC AAT CAG GCA GGA GA
R	TTG GGC TGA GAC GAT GGG GT

L1 reporter (pJJ101-L1; pL1Hs)	
F	CAC CCT AAC TGA CAC ACA TTC CAC
R	CAT GTC TGG ATC CGG CCT CCC

Hygromycin^{R}	
F	CCT GAA CTC ACC GCG ACG TC
R	GTC AAG CAC TTC CGG AAT CGG

GAPDH	
F	GCA AAT TCC ATG GCA CCG T
R	TCG CCC CAC TTG ATT TTG G

pYX014 Renilla	
F	CTT AGG CAG ATC GTC GCT GG
R	CTC CCA AGC AAG ATC ATG CGG

L1 reporter (pYX014; L1-luc)	
F	GGA TGA TCT GGT TGC CGA AG
R	CCT TCG TGA CTT CCC ATT TGC C

L1 LEAP	
F	GGA ATT CGA TGG GCA ATG TGC ACA TGT AC
R	GCG AGC ACA GAA TTA ATA CGA CT

MALAT1	
F	CAT TCG CTT AGT TGG TCT AC
R	TTC TAC CGT TTT TAG CTT C

cMYC	
F	CCT ACC CTC TCA ACG ACA GCA G
R	CTT GTT CCT CCT CAG AGT CGC TG

cJUN	
F	GAT AAT CCA GTC CAG CAA CGG
R	GTT CTG GCT GTG CAG TTC G

PSMB6	
F	CAA CCA CTG GGT CCT ACA TCG C
R	GTT CAA TGC TGT GGA AAC CGA GC

spike in (firefly luciferase)	
F	AAG GTT GTG GAT CTG GAT AC
R	GAT TGT TTA CAT AAC CGG AC

MDM2	
F	GGT TGA CTC AGC TTT TCC TCT TG
R	GGA AAA TGC ATG GTT TAA ATA GCC

Supplementary Table 2. Primers used for qPCR or RT-PCR.

5' adapter	5′-GUU CAG AGU UCU ACA GUC CGA CGA UCN NNN-3′
3' adapter	5′-rApp NN NNT GGA ATT CTC GGG TGC CAA GG/3ddC/-3′
Reverse transcription primer	5′-GCC TTG GCA CCC GAG AAT TCC A-3′
PCR forward primer (RP1)	5′-AAT GAT ACG GCG ACC ACC GAG ATC TAC ACG TTC AGA GTT CTA CAG TCC GA-3′
PCR index reverse primer (RPI X)	5′-CAA GCA GAA GAC GGC ATA CGA GAT **NNN NNN** GTG ACT GGA GTT CCT TGG CAC CCG AGA ATT CCA-3′

RPI X	6 nt index
RPI1	CGTGAT
RPI10	AAGCTA
RPI11	GTAGCC
RPI12	TACAAG

Supplementary Table 3. Oligonucleotides for the construction of pL1Hs-transfected HeLa MeRIP-seq cDNA library.

The sequences of adaptors and primers were designed to ensure compatibility with the Illumina sequencing platform.

Reference

1 Meyer, K. D. *et al.* Comprehensive analysis of mRNA methylation reveals enrichment in 3' UTRs and near stop codons. *Cell* **149**, 1635-1646, doi:10.1016/j.cell.2012.05.003 (2012).
