# Supplementary material for: L1 retrotransposons exploit RNA m6A modification as an evolutionary driving force
Source: Nat Commun. 2021 Feb 9;12:880. doi: 10.1038/s41467-021-21197-1 (PMC7873242; doi:10.1038/s41467-021-21197-1)
Supplement: Supplementary file 6 — Reporting Summary [file 41467_2021_21197_MOESM6_ESM.pdf]

nature research

Corresponding author(s): Kyudong Han and Kwangseog Ahn

Last updated by author(s): Jan 11, 2021

Reporting Summary

Nature Research wishes to improve the reproducibility of the work that we publish. This form provides structure for consistency and transparency in reporting. For further information on Nature Research policies, see our Editorial Policies and the Editorial Policy Checklist.

Statistics

For all statistical analyses, confirm that the following items are present in the figure legend, table legend, main text, or Methods section.

n/a	Confirmed	
☐	☒	The exact sample size (*n*) for each experimental group/condition, given as a discrete number and unit of measurement
☐	☒	A statement on whether measurements were taken from distinct samples or whether the same sample was measured repeatedly
☐	☒	The statistical test(s) used AND whether they are one- or two-sided<br>*Only common tests should be described solely by name; describe more complex techniques in the Methods section.*
☒	☐	A description of all covariates tested
☐	☒	A description of any assumptions or corrections, such as tests of normality and adjustment for multiple comparisons
☐	☒	A full description of the statistical parameters including central tendency (e.g. means) or other basic estimates (e.g. regression coefficient) AND variation (e.g. standard deviation) or associated estimates of uncertainty (e.g. confidence intervals)
☐	☒	For null hypothesis testing, the test statistic (e.g. *F*, *t*, *r*) with confidence intervals, effect sizes, degrees of freedom and *P* value noted<br>*Give P values as exact values whenever suitable.*
☒	☐	For Bayesian analysis, information on the choice of priors and Markov chain Monte Carlo settings
☒	☐	For hierarchical and complex designs, identification of the appropriate level for tests and full reporting of outcomes
☒	☐	Estimates of effect sizes (e.g. Cohen's *d*, Pearson's *r*), indicating how they were calculated

Our web collection on statistics for biologists contains articles on many of the points above.

Software and code

Policy information about availability of computer code

Data collection	For retrotransposition assay, retrotransposition-positive/blasticidin S- resistant colonies were counted using Colony, version 1.1 (Fujifilm).<br>Immunoblot assay images were collected using FusionCapt Advance Solo 4 16.15 (VILBER).<br>Fluorescence microscopic images were acquired using NIS-Elements (Nikon).<br>Northern blot images were acquired using Typhoon™ FLA 7000, version 1.2 (GE healthcare Life sciences)<br>For Annexin V assay, flow cytometry data were analyzed using FlowJo, version 10 (FlowJo)
Data analysis	Bioinformatic analyses (MeRIP, eIF3 PAR CLIP) were performed using Cutadapt (version 2.10), STAR (version 2.5.3a), MACS2 (version 2.1.0), FASTX Toolkit (version 0.0.13.2), and bowtie2 (version 2.2.4).<br>m6A sites were predicted using SRAMP (2016 updated version, http://www.cuilab.cn/sramp/).<br>Fluorescence microscopic images and co-localization analyses were performed using TrackNTrace (version 1.03) and ImageJ (version 1.53).<br>Comparative analyses of species-specific L1s were performed using BLAT (version:human (GRCh37/hg19; Feb. 2009), chimpanzee (CSAC Pan_troglodytes-3.0/panTro5; May. 2016), gorilla (GSMRT3/gorGor5; May. 2016)), RepeatMasker (version 4.1.0), MUSCLE (version 3.8.31, https://www.ebi.ac.uk/Tools/msa/muscle/), and Weblogo (version 2.8.2, https://weblogo.berkeley.edu/logo.cgi).<br>Statistical analysis was performed using GraphPad Prism 7 software.

For manuscripts utilizing custom algorithms or software that are central to the research but not yet described in published literature, software must be made available to editors and reviewers. We strongly encourage code deposition in a community repository (e.g. GitHub). See the Nature Research guidelines for submitting code & software for further information.

Data

Policy information about availability of data

All manuscripts must include a data availability statement. This statement should provide the following information, where applicable:

- Accession codes, unique identifiers, or web links for publicly available datasets
- A list of figures that have associated raw data
- A description of any restrictions on data availability

MeRIP-seq data from pL1-expressing HeLa cells have been deposited in GEO(GSE152328). All source data will be made available upon request.

Field-specific reporting

Please select the one below that is the best fit for your research. If you are not sure, read the appropriate sections before making your selection.

☒ Life sciences ☐ Behavioural & social sciences ☐ Ecological, evolutionary & environmental sciences

For a reference copy of the document with all sections, see nature.com/documents/nr-reporting-summary-flat.pdf

Life sciences study design

All studies must disclose on these points even when the disclosure is negative.

Sample size	Sample size was determined based on previosuly published studies (ref. 22, 23, 25, 34) and on experience and was equivalent to that is routinely used for any particular assay. All experiments were executed in independent biological replicates and individual replicate numbers are given in the figure legends. For L1 retrotransposition assays, luciferase assays, LEAP and qPCR analyses, at least 3 independent experiments were carried out. Immunoblot images are representative of at least three independent experiments in figure 1E-G, 4A,C and 6D. Immunoblot images in supplementary information figure are representative of at least two independent experiments. MeRIP seq were performed in two independent experiments. Viability assay and quantification of transfection efficiency were performed twice independently.
Data exclusions	No data were excluded from the analysis.
Replication	Most experiments are reliably reproduced in two or more independent experiments. The number of replications for each experiment are indicated in figure legend respectively.
Randomization	No randomization was required because the study was based on molecular and cellular biology techniques.
Blinding	The investigators who carried out the experiments were not blinded to the groups and we did not consider blinding necessary because all groups were treated in unbiased manner by algorithms and computer programs.

Reporting for specific materials, systems and methods

We require information from authors about some types of materials, experimental systems and methods used in many studies. Here, indicate whether each material, system or method listed is relevant to your study. If you are not sure if a list item applies to your research, read the appropriate section before selecting a response.

Materials & experimental systems

n/a	Involved in the study	
☐	☒	Antibodies
☐	☒	Eukaryotic cell lines
☒	☐	Palaeontology and archaeology
☒	☐	Animals and other organisms
☒	☐	Human research participants
☒	☐	Clinical data
☒	☐	Dual use research of concern

Methods

n/a	Involved in the study	
☒	☐	ChIP-seq
☐	☒	Flow cytometry
☒	☐	MRI-based neuroimaging

Antibodies

Antibodies used	Anti-LINE-1 ORF1p Antibody, clone 4H1 (Merck, MABC1152)<br>Anti METTL3 antibody (Abcam, ab195352)<br>Anti ALKBH5 antibody (Novus, NBP1-82188)<br>Anti FTO antibody (Abcam, ab124892)<br>Anti-N6-methyladenosine (m6A) Antibody (Merck, ABE572)<br>Anti Vinculin antibody (Sigma, V9131)<br>Anti HSP70 antibody (Stressgen, SPA-810)

ANTI-FLAG® M2 antibody (Sigma, F3165)
Anti-T7 tag® antibody (Abcam, ab9138)
Anti eIF3b antibody (Bethyl, A301-761A)
Anti-pan Ago Antibody, clone 2A8 (Merck, MABE56)
Anti GAPDH antibody (AbFrontier, LF-PA0212)
Anti GFP antibody (Santa Cruz Biotechnology, SC-9996)
Anti HA antibody (Cell signaling, 3724)
Peroxidase AffiniPure Goat Anti-Mouse IgG (Jackson ImmunoResearch Laboratories, 115-035-062)
Peroxidase AffiniPure Goat Anti-Rabbit IgG (Jackson ImmunoResearch Laboratories, 111-035-003)
Fluorescein (FITC) AffiniPure Rabbit Anti-Goat IgG (Jackson ImmunoResearch Laboratories, 305-095-047)

Validation

All antibodies are commercially available. Antibodies were validated to react with the human cell-produced proteins by the manufacturer. Additionally, L1 ORF1p, METTL3, ALKBH5, and FTO antibodies were validated with in our own experimental model using RNAi-mediated knockdown.

Eukaryotic cell lines

Policy information about cell lines

Cell line source(s)

HeLa cells were provided by Dr. V Narry Kim (Seoul National University). PA-1 cells were obtained from Yong-Sang Song (Seoul National University). HeLa cells and PA-1 cells were commercially availabe in ATCC (HeLa; ATCC CCL-2, PA-1; ATCC CRL-1572). H9 hESCs were purchased from Wicell Research.

Authentication

HeLa cells were authenticated by ATCC cell authentication using the short tandem repeat (STR) method.

Mycoplasma contamination

HeLa cells and PA-1 cells were confirmed mycoplasma-free by COSMOGENETECH.

Commonly misidentified lines
(See ICLAC register)

No commonly misidentified cell lines were used.

Flow Cytometry

Plots

Confirm that:

☒ The axis labels state the marker and fluorochrome used (e.g. CD4-FITC).

☒ The axis scales are clearly visible. Include numbers along axes only for bottom left plot of group (a 'group' is an analysis of identical markers).

☒ All plots are contour plots with outliers or pseudocolor plots.

☒ A numerical value for number of cells or percentage (with statistics) is provided.

Methodology

Sample preparation

Cells were trypsinized, resuspended in culture media and washed and resuspended in PBS. Cells were labeled with 5 µl of APC-conjugated Annexin V (BioLegend, 640920) according to the manufacturer's instructions. After incubation in the dark for 20 min at RT, the cells were analyzed by flow cytometry using Flow-Activated Cell Sorter Canto II (BD Bioscience) and Flowjo software (version 10, Flowjo).

Instrument

Flow-Activated Cell Sorter Canto II (BD Bioscience)

Software

Flowjo software (version 10, Flowjo)

Cell population abundance

All flow cytometry analysis data were obtained from 10,000 cells counted.

Gating strategy

For all experiments, live cells were gated on by excluding debris, based on the forward and side scatter profiles. A gate of APC positive cells was made using the negative control sample and copied to all samples.The AnnexinV-APC-negative population was regarded as alive, whereas the Annexin V-APC–positive populations were taken as measurements of apoptotic/necrotic cells. The gating strategy is provided in the Figure S1 of the Source data file.

☒ Tick this box to confirm that a figure exemplifying the gating strategy is provided in the Supplementary Information.
